# dTip60 HAT Activity Controls Synaptic Bouton Expansion at the *Drosophila* Neuromuscular Junction

**DOI:** 10.1371/journal.pone.0026202

**Published:** 2011-10-27

**Authors:** Jessica Sarthi, Felice Elefant

**Affiliations:** Department of Biology, Drexel University, Philadelphia, Pennsylvania, United States of America; French National Centre for Scientific Research, France

## Abstract

**Background:**

Histone acetylation of chromatin plays a key role in promoting the dynamic transcriptional responses in neurons that influence the neuroplasticity linked to cognitive ability, yet the specific histone acetyltransferases (HATs) that create such epigenetic marks remain to be elucidated.

**Methods and Findings:**

Here we use the *Drosophila* neuromuscular junction (NMJ) as a well-characterized synapse model to identify HATs that control synaptic remodeling and structure. We show that the HAT dTip60 is concentrated both pre and post-synaptically within the NMJ. Presynaptic targeted reduction of dTip60 HAT activity causes a significant increase in synaptic bouton number that specifically affects type Is boutons. The excess boutons show a suppression of the active zone synaptic function marker bruchpilot, suggesting defects in neurotransmission function. Analysis of microtubule organization within these excess boutons using immunohistochemical staining to the microtubule associated protein futsch reveals a significant increase in the rearrangement of microtubule loop architecture that is required for bouton division. Moreover, α-tubulin acetylation levels of microtubules specifically extending into the terminal synaptic boutons are reduced in response to dTip60 HAT reduction.

**Conclusions:**

Our results are the first to demonstrate a causative role for the HAT dTip60 in the control of synaptic plasticity that is achieved, at least in part, *via* regulation of the synaptic microtubule cytoskeleton. These findings have implications for dTip60 HAT dependant epigenetic mechanisms underlying cognitive function.

## Introduction

Synaptic plasticity, or activity dependent changes in synaptic strength, is the mechanism by which information is processed, and stored as short or long term memory in the central nervous system (CNS) [1], [2], [3]. Epigenetic regulation of chromatin structure plays a key role in providing a coordinated system of gene control critical for promoting the distinct phases of neuronal plasticity that are subsequently converted to short and long term memory formation [4], [5]. One such epigenetic modification is histone acetylation, that serves to epigenetically mark DNA associated histone proteins within chromatin at distinct sites and patterns over time to drive gene expression profiles in the brain [6], [7], [8], [9], [10]. Recent studies support the concept that aberrant changes to the histone acetylation code within the genome of the aging brain cause gene misregulation that drives cognitive decline [11], [12], [13], [14], [15]. As individuals age, the accumulation of inappropriate changes in these epigenetic marks is thought to alter transcription of synaptic plasticity genes with subsequent negative consequences on cognitive function [11], [16], [17]. Although the histone acetyltransferase activity of CREB binding protein (CBP) has been implicated in synaptic plasticity linked gene regulation, additional specific histone acetyltransferases (HATs) important in these processes remain to be elucidated [5], [13], [18].

Work from our laboratory support a role for the HAT Tip60 in nervous system function [19] The Tat-interactive protein-60 kDa (Tip60) is a member of the MYST family of histone acetyltransferases (HATs) [20]. Tip60 plays essential roles in many cellular processes in large part, by its function in regulating gene expression profiles *via* histone acetylation [21], [22]. We previously demonstrated that the *Drosophila* homolog of mammalian Tip60 (dTip60) is produced robustly in the anterior brain neuroblast population regions of the central nervous system (CNS) in *Drosophila*, *in vivo*, suggesting a role for dTip60 in synapse formation in the brain. Moreover, our microarray analysis of flies depleted in dTip60 HAT activity reveal that dTip60 regulates genes enriched for neuronal functions that include neuronal development and synaptic function [19]. These findings prompted us to investigate a causative role for dTip60 in synaptic plasticity, with the rationale that such findings may have implications for this HAT in cognitive ability.

In this report, we ask whether the HAT Tip60 plays a causative role in the control of synaptic plasticity and structure using the *Drosophila* neuromuscular junction (NMJ) as a well characterized neuroplasticity model [23]. By analyzing the effects of dTip60 HAT activity misregulation on synaptic growth, we causatively link dTip60 HAT activity in negatively controlling synaptic bouton formation *via* regulation of the synaptic microtubule cytoskeleton. Our results are the first to demonstrate a novel role for dTip60 in the control of synaptic plasticity at the *Drosophila* NMJ.

## Results

### dTip60 is localized at the pre and postsynaptic sides of *Drosophila* NMJ

**Figure 1:**
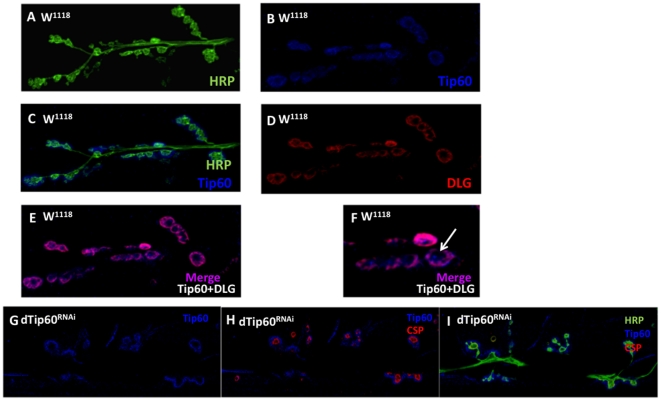
dTip60 is localized at the pre and postsynaptic sides of larval NMJ. Figures (**A–E**) represent confocal imaging analysis of control w^1118^ larval boutons on muscles 6/7 at abdominal segment A4 immunohistochemically triple stained with (**A**) HRP antibody (green) that labels the entire presynaptic membrane (**B**) dTip60 antibody (blue) and (**C**) HRP and dTip60 merged (green and blue) (**D**) Dlg antibody stain (red) that labels post-synaptic membrane. (**E**) dTip60 and Dlg merged (purple) image showing dTip60 co-localization with post-synaptic labeled Dlg-membrane and dTip60 immunoreactivity within the presynaptic bouton. (**F**) Enlargement of Figure (**E**) where arrow depicts dTip60 immunoreactivity within the presynaptic bouton. Figures (**G–I**) represent confocal imaging analysis of presynaptic RNAi knockdown of dTip60 in larval boutons on muscles 6/7 at abdominal segment A4 immunohistochemically triple stained with pre-synaptic markers HRP, cysteine string protein (csp) antibody (red), a presynaptic vesicle protein that controls vesicle exocytosis and Tip60 (blue). (**G**) dTip60 (blue) showing the presence of postsynaptic dTip60 and absence of presynaptic dTip60 localization due to RNAi presynaptic knockdown demonstrating specificity of dTip60 Ab and confirming dTip60 pre and postsynaptic localization (**H**) dTip60 and Csp merged (blue and red) and (**I**) dTip60, Csp and HRP merged (blue, green and red). In the analyses, wild-type control w^1118^ genotype and test dTip60^RNAi^ genotypes are each represented by 25 larval preparations (n = 25). Scale bar 10 um.

Our previous microarray analysis of mutant flies specifically depleted for dTip60 HAT activity identified misregulated genes enriched for diverse neuronal processes, many of which were linked to synaptic function [19]. These findings prompted us to ask whether Tip60 was localized at the synapses of the *Drosophila* NMJ. The *Drosophila* NMJ is a dynamic structure that constantly changes in response to activity and body size, making it a particularly suitable model to investigate proteins involved in synaptic plasticity [24], [25], [26]. Moreover, it provides relevance for understanding brain function in that it shares central features with major excitatory synapses of the mammalian brain including ionotropic glutamate receptors and many other proteins also found in mammalian central synapses [24], [27], [28]. To visualize proteins localized in the NMJ, we performed immunohistochemistry using antibodies to dTip60 and antibodies against HRP, a commonly used marker that specifically labels the entire presynaptic membrane [29], [30] (Figure 1, A–C). These studies revealed enrichment of dTip60 that was concentrated at the synapses of the NMJ and overlapped with preysnaptic marker HRP, indicating that dTip60 was presynpatically localized. Co-localization studies using antibodies to Tip60 and the well characterized and commonly used postsynaptic density marker protein Discs Large (Dlg) [29], [31] revealed co-localization of dTip60 with Dlg (Figure 1, D–F). Importantly, these images also showed dTip60 immunoreactivity within the presynaptic boutons in a pattern identical to presynaptic HRP staining (Figure 1F), supporting a pre and postsynaptic localization of dTip60 in the NMJ boutons. To confirm pre and postsynaptic localization of dTip60 and the specificity of the dTip60 Ab at the NMJ, we knocked down dTip60 levels specifically in the presynaptic NMJ using the GAL4/UAS targeted gene expression system with well characterized fly lines we previously created that carry a UAS responsive transgene for dTip60^RNAi^ [29], [30]. This fly line was crossed to the pan-neuronal presynaptic driver GAL4 driver elav^C155^ and localization and levels of dTip60 were assessed using immunohistochemistry with antibodies against dTip60 and another presynaptic marker cysteine string protein (csp) (Figure 1 G–I). These studies revealed the presence of postsynaptic dTip60 and importantly, the absence of presynaptic dTip60 localization due to RNAi presynaptic knockdown, demonstrating specificity of the dTip60 Ab at the NMJ and confirming dTip60 pre and postsynaptic localization. Post-synaptic knockdown of dTip60 using GAL4 driver MEF-2 showed reduction of dTip60 postsynaptic localization, further confirming pre and postsynaptic localization of dTip60 and the efficacy of RNAi induced dTip60 knockdown (Figure S1). Taken together, these results demonstrate that dTip60 is localized at both the pre and postsynaptic sides of the *Drosophila* NMJ, suggesting a role for dTip60 in synaptic function.

### Pre and postsynaptic dTip60 HAT activity controls synaptic bouton formation in the developing NMJ

The enrichment of dTip60 in both pre and postsynaptic sides of the NMJ prompted us to investigate a potential function for dTip60 in bouton development by examining the effects resulting from depletion or overexpression of dTip60 specifically in the nervous system. Misregulation of dTip60 was achieved for all experiments by utilizing the GAL4/UAS targeted gene expression system with flies carrying UAS responsive transgenes for either dTip60^RNAi^, a dominant negative HAT defective version of dTip60 (dTip60^E431Q^), or wild-type dTip60 (dTip60^WT^), all well characterized transgenic fly lines created in our laboratory [19], [32]. Independent fly line dTip60^RNAi^ A was chosen for this study as it is our strongest dTip60^RNAi^ line [32] and showed robust knockdown of dTip60 pre and postsynaptically (Figure 1G–I). Independent fly lines dTip60^E431Q^ line B and dTip60^WT^ line B were selected for this study as these lines show equivalent and robust expression of transgene expression as assessed by qPCR [19] as well as equivalent Gal4 driven expression at the NMJ as assessed by immunostaining using dTip60 Abs (Figure S1). Of note, Tip60^RNAi^ and Tip60^E431Q^ induced lethality can be fully rescued by specifically increasing dTip60 levels [19], [32], demonstrating that the phenotypic effects we observe in our Tip60 mutants are indeed specifically caused by disruption of Tip60 HAT function.

**Figure 2:**
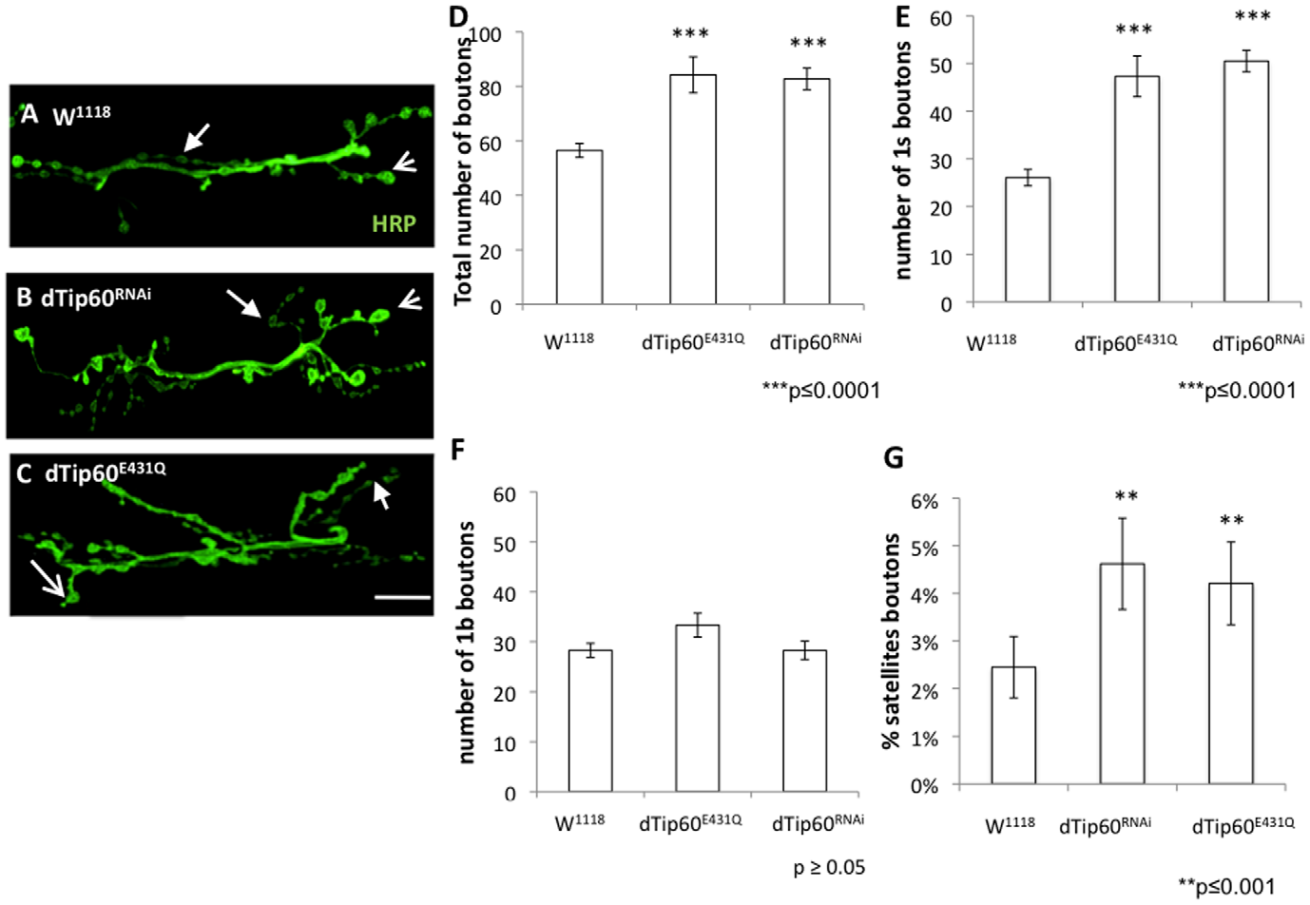
Presynaptic reduction of dTip60 in the nervous system leads to an expansion of synaptic boutons at larval NMJs. Flies homozygous for either dTip60^E431Q^, dTip60^RNAi^ or control w^1118^ were crossed to flies homozygous for the nervous system elav^C155^ pan-neuronal GAL4 driver, and staged third instar progeny larvae were collected. Confocal imaging analysis of larval boutons on muscles 6/7 at abdominal segment A4 immunohistochemically stained with anti-HRP (green) that labels the entire presynaptic membrane. (**A**) control w^1118^ larvae (**B**) larvae expressing dTip60^RNAi^ (**C**) larvae expressing dTip60^E431Q^. Scale bar 10 um. Line arrow depicts Ib bouton, thick arrow depicts Is bouton. Histogram depicts quantitative analysis of bouton number on muscles 6 and 7 at abdominal segment 4 where (**D**) represents total bouton number (**E**) number of Is boutons (**F**) number of Ib boutons and (**G**) percentage of satellite boutons. Small arrow depicts type Is bouton, line arrow depicts type Ib bouton. In the analyses, w^1118^ genotype is represented by 18 larval preparations (n = 18), dTip60^RNAi^ (n = 19) and dTip60^E431Q^ (n = 19). Asterisks (*) indicates statistically significant difference in relation to control where single asterisks indicate p≤0.001 and double asterisks indicate p≤0.0001. All error bars depict standard error of the mean.

To investigate a presynaptic role for dTip60 in bouton formation, fly lines dTip60^RNAi^, dTip60^E431Q^, dTip60^WT^, and w^1118^ control flies were each crossed to the presynaptic pan-neuronal driver GAL4 driver elav^C155^. Induction of dTip60^RNAi^ and dTip60^E431Q^ resulted in pupal lethality while dTip60^WT^ and w^1118^ control flies showed no observable negative phenotypic effects. To examine basic synaptic morphology, boutons at muscles 6 and 7 at abdominal segment A4 were stained with anti-HRP antibodies that label and allow for visualization of the entire presynaptic membrane, and Phalloidin, a toxin that stains F-actin within the muscles, to identify and measure the surface area of the appropriate muscle groups and abdominal segments (data not shown). Changes in NMJ development were assessed by counting the number of synaptic boutons for each genetic strain. This analysis revealed that reduction of dTip60 had clear consequences on the expansion of the boutons, while muscle surface area remained unchanged. Remarkably, there was a significant increase of the total number of synaptic boutons in the dTip60^RNAi^ and dTip60^E431Q^ larvae (56.45±2.54 for w^1118^, 84.26±6.06 for Tip60^E431Q^ and 82.73±3.97 for Tip60^RNAi^) when compared with the wild type control (Figure 2A–D; all error bars are standard error of mean). Of note, there are two types of neurons that innervate the larval NMJ at muscles 6/7 giving rise to two sets of boutons. These boutons are classified as type I small (Is) and type I big (Ib) based on their size. Type Is boutons have larger stimulation thresholds and excitatory junctional currents of larger amplitude while type Ib boutons exhibit more pronounced short-term facilitation [25], [33]. Intriguingly, although the total number of boutons in the dTip60^RNAi^ and dTip60^E431Q^ lines were significantly increased when compared to the wild-type control, there was a substantially larger expansion of type-Is boutons when directly compared to Ib (26.05±1.7 type Is and 28.8±1.4 type Ib for w^1118^, 50.52±2.2 type Is boutons and 28.26±1.8 type Ib boutons for dTip60^RNAi^, 47.31±4.2 type Is and 33.31±2.4 type Ib for dTip60^E431Q^). Thus, in comparison to the w^1118^ control, there was a 92.82% increase in type Is and 0% increase in type Ib for dTip60^RNAi^, and a 80.59% increase in type Is and a 15.65% increase in type Ib for dTip60^E431Q^. Additionally, “satellite” bouton budding, a process that involves the budding of bouton(s) from one central parent bouton on the main branch to form smaller “satellites boutons”, was also affected in response to dTip60 reduction. We observed a significant increase in the number of satellite boutons in the dTip60^RNAi^ and dTip60^E431Q^ when compared to the w^1118^ (2.45%±0.64 for w^1118^, 4.62%±0.96 for Tip60^RNAi^, 4.21%±0.87% for Tip60^E431Q^, Figure 2I). Finally, we observed no significant changes in bouton number in dTip60^WT^ flies when compared to the control flies, consistent with our observation that induction of dTip60^WT^ in the nervous system led to no observable phenotypic effects and no significant effects on gene expression in our prior microarray analysis [19]. Of note, bouton counts for UAS-RNAi dTip60 control flies that contain a non-inverted RNAi target sequence [32], all UAS lines in the absence of the GAL4 driver, and GAL4 driver in the absence of the UAS transgene showed no significant changes with respect to the w^1118^ control flies. Moreover, bouton counts for additional independent fly lines dTip60^WT^ line A and dTip60^E431Q^ line A crossed to the GAL4 driver elav^c155^ showed similar bouton counts to dTip60^WT^ line B and dTip60^E431Q^ line B, respectively. These additional controls, as well as our observation that both dTip60^E431Q^ and dTip60^RNAi^ showed similar changes in bouton number, confirmed that changes in bouton number were indeed due to disruption of dTip60 at the NMJ (Figure S2 A and B).

**Figure 3:**
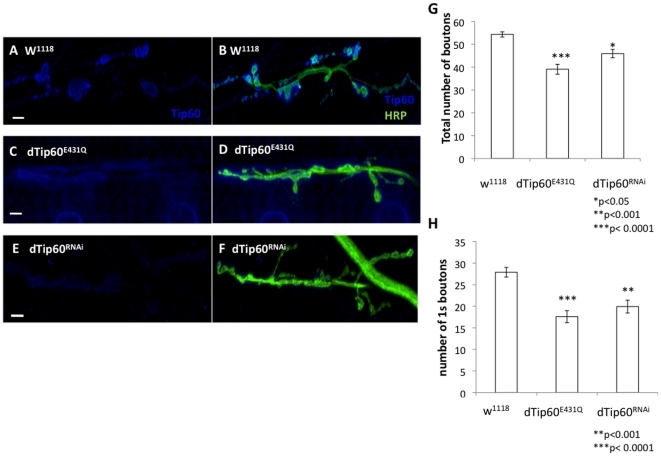
Postsynaptic reduction of dTip60 in the nervous system leads to a decrease of synaptic boutons at larval NMJs. Flies homozygous for either dTip60^E431Q^, dTip60^RNAi^ or control w^1118^ were crossed to flies homozygous for the muscle specific GAL4 driver MEF-2, and staged third instar progeny larvae were collected. Confocal imaging analysis of larval boutons on muscles 6/7 at abdominal segment A4 double labeled with anti-HRP (green) that labels the entire presynaptic membrane and anti-dTip60 antibody (blue). (**A and B**) control w^1118^ larvae (**C and D**) larvae expressing dTip60^E431Q^ (**E and F**) larvae expressing dTip60^RNAi^. Scale bar 10 um. Line arrow depicts Ib bouton, thick arrow depicts Is bouton. Histogram depicts quantitative analysis of bouton number on muscles 6 and 7 at abdominal segment 4 where (**G**) represents total bouton number (**H**) number of Is boutons. In the analyses, w^1118^ genotype is represented by 18 larval preparations (n = 18), dTip60^RNAi^ (n = 19) and dTip60^E431Q^ (n = 19). Asterisks (*) indicates statistically significant difference in relation to control where single asterisks indicate p≤0.001 and double asterisks indicate p≤0.0001. All error bars depict standard error of the mean.

Our observation that dTip60 is localized both pre and postsynaptically, prompted us to ask whether dTip60 also plays a postsynaptic role in regulating bouton formation at the NMJ. For this analysis, fly lines dTip60^RNAi^, dTip60^E431Q^, dTip60^WT^, and w^1118^ control flies were each crossed to the postsynaptic muscles specific GAL4 driver MEF-2 and quantitation of bouton number at the NMJ was carried out as described for our presynaptic analysis. Similar to presynaptic knockdown of dTip60, postsynaptic induction of dTip60^RNAi^ and dTip60^E431Q^ resulted in pupal lethality while dTip60^WT^ and w^1118^ control flies showed no observable negative effects on viability. However, quantitative bouton analysis revealed that in contrast to the increased number of boutons we observed in response to presynaptic dTip60 loss (Figure 2), here we observed a significant reduction in bouton number in both dTip60^RNAi^ and dTip60^E321Q^ larvae that was specific for 1s boutons (total number of boutons were 54.33±1.14 for w^1118^, 39.08±2.17 for dTip60^E431Q^, 45.92±1.83 for dTip60^RNAi^, 1s bouton numbers were 27.8±1.11 for w^1118^, 17.58±1.4 for dTip60^E431Q^, 19.92±1.49 for dTip60^RNAi^) as well as the absence of satellite boutons (Figure 3). Of note, no significant changes in bouton number were observed in dTip60^WT^ larvae when compared to the w^1118^ control larvae. Analysis using a second muscle specific driver GAL4 twi;mef2 showed similar results (data not shown). Taken together, our results indicate that dTip60 plays both pre and postsynaptic roles in controlling the degree of synaptic bouton number, and displays at least some presynaptic specificity in preferentially controlling type Is bouton development.

### Presynaptic reduction of dTip60 in the nervous system leads to suppression of active zone marker bruchpilot at larval NMJs

**Figure 4:**
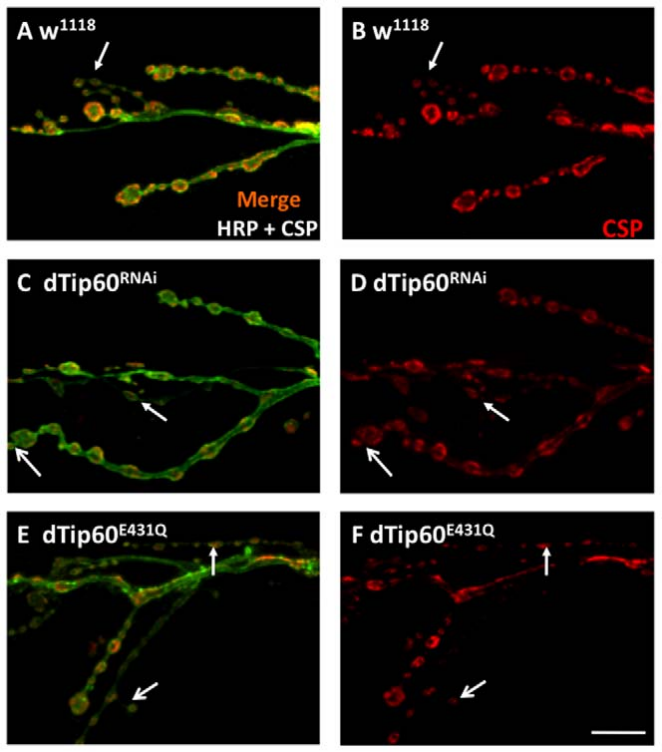
Presynaptic vesicle protein distribution is not altered in dTip60 mutants. Confocal imaging analysis of larval boutons on muscles 6/7 at abdominal segment A4 immunohistochemical double labeled with HRP antibody (green) that labels the entire presynaptic membrane and cysteine string protein (csp) antibody (red), a presnynaptic vesicle protein that controls vesicle exoctyosis. (**A,B**) wild-type control w^1118^ (**C,D**) dTip60^RNAi^ (**E,F**) dTip60^E431Q^. Immunostaining with csp antibodies indicate that these vesicle proteins are present in mutant dTip60 NMJs with a distribution similar to wild-type. In the analyses, w^1118^ genotype is represented by 21 larval preparations (n = 21), dTip60^RNAi^ (n = 19) and dTip60^E431Q^ (n = 20). Line arrow depicts Is bouton, small thick arrow depicts satellite bouton. Scale bar is 10 um.

The effects of dTip60 reduction on NMJ expansion led us to ask whether the additional synaptic boutons that were formed in response to presynaptic dTip60 reduction impacted the production of synaptic machinery. To address this question, we analyzed the presence and distribution of two presynaptic vesicle associated proteins essential for synaptic function: the cysteine string protein (csp) that regulates the activity of presynaptic Ca^2+^ channels to control exocytosis, and synaptotagmin (syt), a protein that promotes both synaptic vesicle fusion and endocytosis mediated vesicle recycling [34], [35], [36], thus functionally “marking” both sides of the vesicle cycle. Immunostainings against these proteins revealed that type Is, Ib and satellite boutons in dTip60^RNAi^ and dTip60^E431Q^ flies were immunoreactive for these markers (Figure 4 A–F shows csp stain; data not shown for syt stain), and that the intensity and distribution of these proteins was identical as compared to wild-type w^1118^ control flies. These results suggested normal morphology and functionality for these particular vesicle proteins in synaptic transmission.

**Figure 5:**
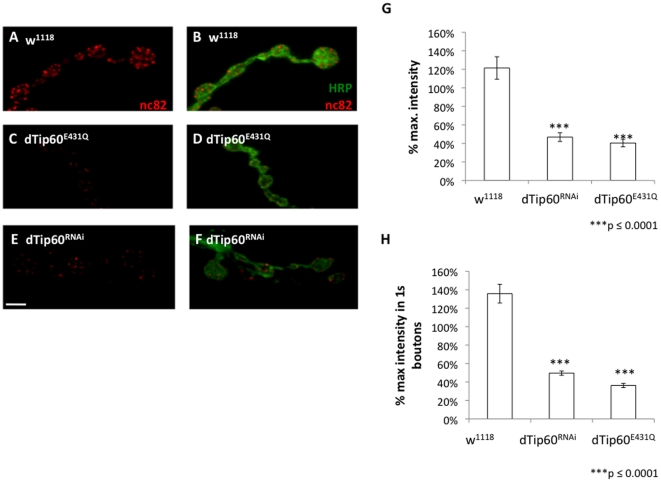
Presynaptic reduction of dTip60 in the nervous system leads to suppression of active zone marker bruchpilot at larval NMJs. Confocal imaging analysis of larval boutons on muscles 6/7 at abdominal segment 4 immunohistochemical double labeled with HRP antibody (green) that labels the entire presynaptic membrane and nc82 antibody (red) that recognizes bruchpilot, a protein associated with functional active zones and neurotransmission. (**A,B**) is w^1118^ (**C,D**) is dTip60^E431Q^ (**E,F**) is dTip60^RNAi^. In the analyses, w^1118^ genotype is represented by 20 larval preparations (n = 20), dTip60^RNAi^ (n = 18) and dTip60^E431Q^ (n = 21). Scale bar is 5 um. Quantification of the fluorescence intensity as a measure of nc82 abundance at the NMJ muscle 6/7 of segment A4. The relative fluorescence intensities were measured as number of pixels per measured area and represented as % maximum intensities. Fluorescence intensity was determined by first quantitatively measuring the intensity profiles of all the nc82 punctae in a given marked area of the boutons and then calculating the average intensity of the brightest punctae in all of the samples as given by their intensity values. The nc82 intensities were normalized against HRP fluorescence intensities and corrected for background before all intensity measurements. Histogram depicts quantitative analysis of antibody fluorescence intensity where (**G**) is percentage maximum intensity of antibody stain in all boutons (**H**) is percentage maximum intensity of antibody stain in Is boutons. Asterisks (*) indicates statistically significant difference in relation to control where double asterisks indicate p≤0.001 and three asterisks indicate p≤0.0001. All error bars depict standard error of the mean.

Bouton number within the fly NMJ is constantly changing to correlate with changes in muscle size as development proceeds. Bouton number changes are accompanied by compensatory changes in the number of synapses, so that synaptic functionality is also maintained throughout development [29], [37]. Thus, it is important to ask whether the expansion in bouton number that we observed in response to dTip60 loss is also accompanied by compensatory changes in synapse number. To address this question, we carried out immunostaining with antibody nc82, a commonly used marker that recognizes the *Drosophila* active zone protein component Bruchpilot (BRP) [24], [38]. Active zones are presynaptic specializations where synaptic vesicles accumulate and fuse to the plasma membrane in response to an action potential, and thus nc82 is a commonly used marker for both synapse number and functionality in synaptic transmission. Both dTip60^RNAi^ and dTip60^E431Q^ larvae showed presence of nc82 staining, confirming the presence of active zones in these dTip60 mutants (Figure 5 A–F). To determine if dTip60 has an impact on the number of active zones within the boutons, we measured the mean fluorescence intensity of nc82 staining which should be proportional to the number of active zones and is a correlate of synaptic function [39]. The percentage maximum intensities for both dTip60^RNAi^ and dTip60^E431Q^ flies were found to be significantly lower when compared to the w^1118^ control line (Figure 5 G,H). Of note, nc82 staining patterns were normal for all UAS fly lines in the absence of the GAL4 transgene. Thus, although there was an increase in bouton number that resulted from dTip60 loss, the boutons show a decrease in the abundance in the active zone synaptic function marker bruchpilot, suggesting potential defects in their neurotransmission function. Taken together, these results demonstrate that Tip60 is required to regulate both bouton number and appropriate production of synaptic transmission machinery during NMJ development.

### dTip60 controls bouton expansion by affecting microtubule cytoskeleton dynamics

Regulated microtubule dynamics and architecture is an essential element in the control of synapse division. The formation of synaptic boutons at axon terminals is achieved *via* microtubules that invade and promote bouton budding at the plasma membrane. [24], [40] Hairpin microtubule loop formation is associated with stable synaptic boutons within the *Drosophila* NMJ, while the opening or splaying of these loops is associated with boutons undergoing division or sprouting. As such, many synaptic bouton NMJ overgrowth phenotypes, particularly those with excess satellite boutons, are also associated with an excess of microtubule loops [29], [41]. Thus, we asked whether the increase in normal and satellite boutons we observed in response to dTip60 reduction was related to alterations in microtubule cytoskeleton dynamics. To address this question, we carried out immunostaining against the *Drosophila* MAP1B homolog Futsch, a microtubule associated protein shown to be essential for the stabilization of microtubule hairpin loop formation that promotes synaptic bouton formation [41], [42]. Accordingly, Futsch mutants lacking Futsch protein show a decrease in bouton numbers and Futsch has been shown to completely colocalize with microtubules only at proximal boutons undergoing division, and is not found at terminal boutons as these boutons have typically completed division [41], [43]. Because of these findings, Futsch is a commonly used marker to depict whether Futsch associated microtubule loops have formed that in turn, promote bouton formation and therefore represent sites of active bouton division [41].

**Figure 6:**
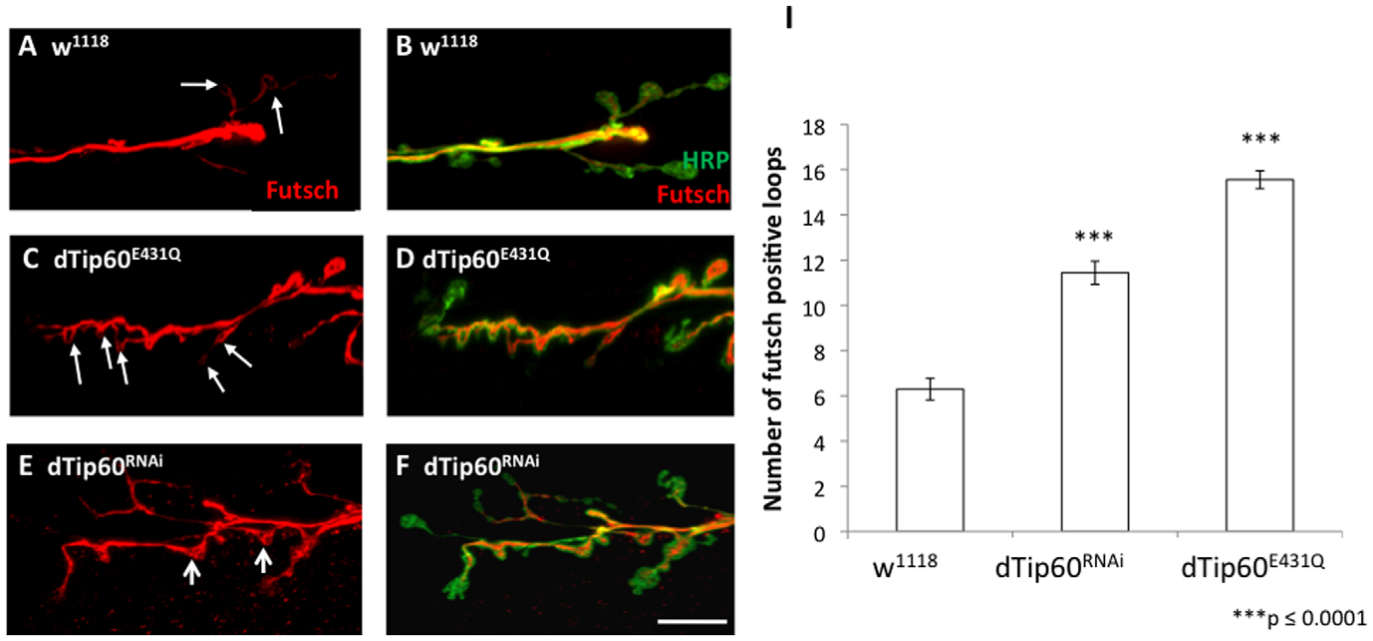
The number of presynatpic futsch-positive loops are increased at synapses in mutant dTip60 larvae. Confocal imaging analysis of larval boutons on muscles 6/7 at abdominal segment 4 immunohistochemical double labeled with HRP antibody (green) that labels the entire presynaptic membrane and Futsch antibody (red) that labels Futsch stained microtubule loops. (**A,B**) is w^1118^ (**C,D**) is dTip60^E431Q^ (**E,F**) is dTip60^RNAi^. Histogram depicts quantitative analysis of Futsch-positive loop number on muscles 6 and 7 at abdominal segment A4 where (**G**) is total number of loops. In the analyses, w^1118^ genotype is represented by 30 larval preparations (n = 30), dTip60^RNAi^ (n = 25) and dTip60^E431Q^ (n = 27). Small thick arrows depict Futsch stained microtubule loops (**C**), line arrows (**E**) depict Futsch stained microtubule rearrangement within loops. Scale bar is 10 um. Asterisks (\) indicates statistically significant difference in relation to control where double asterisks indicate p≤0.001 and three asterisks indicate p≤0.0001. All error bars depict standard error of the mean.

Fly lines dTip60^RNAi^, dTip60^E431Q^ were expressed presynaptically in the motorneurons using the pan-neuronal driver GAL4 driver elav^C155^, and the number of Futsch immunostained microtubule loops was quantified for each genotype. Fly line w^1118^ was used for a control. Presynaptic overexpression of Tip60^E431Q^ showed a drastic and significant increase in the number of futsch loops as compared to the w^1118^ control (Figure 6, A–D). Quantification of the number of loops confirmed this increase (15±0.3 for dTip60^E431Q^, 6±0.4 for w^1118^) (Figure 6 G), consistent with the increased number of boutons we observe (Figure 2). Intriguingly, presynaptic RNAi reduction of dTip60 also revealed a significant increase in futsch positive loops when compared to the control (11±0.5 for Tip60^RNAi^) (Figure 6 E–F), however the increase was less than that of the dTip60^E431Q^ mutants (Figure 6 C–D). Moreover, in contrast to dTip60^E431Q^ flies, the dTip60^RNAi^ mutants showed increased reorganization and splaying of futsch-associated microtubules shown to precede loop formation in newly divided or dividing boutons (Figure 6 G–H). The differences in Futsch associated microtubule reorganization between dTip60^RNAi^ versus dTip60^E431Q^ line may reflect the different mechanisms of dTip60 knockdown between the two lines. For example, dTip60^E431Q^ effects are directly reliant on the HAT activity of dTip60 and its competition with wild-type dTip60, while dTip60 RNAi induced knockdown may also interfere with additional dTip60 processes, such as dTip60 complex formation with additional proteins that carry out various functions [44]. Normalization of bouton number to bouton loops in w^1118^ control, dTip60^RNAi^ and dTip60^E431Q^ larvae revealed that there was also a significant increase in this number in the mutant dTip60 larvae when compared to w^1118^ control (Figure S3). Of note, Futsch staining patterns were similar to that of w^1118^ control larvae for all UAS fly lines in the absence of the GAL4 transgene, indicating that changes in futsch associated microtubule dynamics resulted from loss of dTip60 in the nervous system. Thus, changes in dTip60 levels via RNAi knockdown or dTip60 HAT activity by dominant negative dTip60^E431Q^ production at the presynaptic level both affect the organization of microtubule architecture, albeit at different levels, *via* Futsch associated mechanisms, with consequences on synaptic bouton division and formation.

### Loss of presynaptic dTip60 in the nervous system results in a reduction of acetylated microtubules in axons extending into terminal boutons

**Figure 7:**
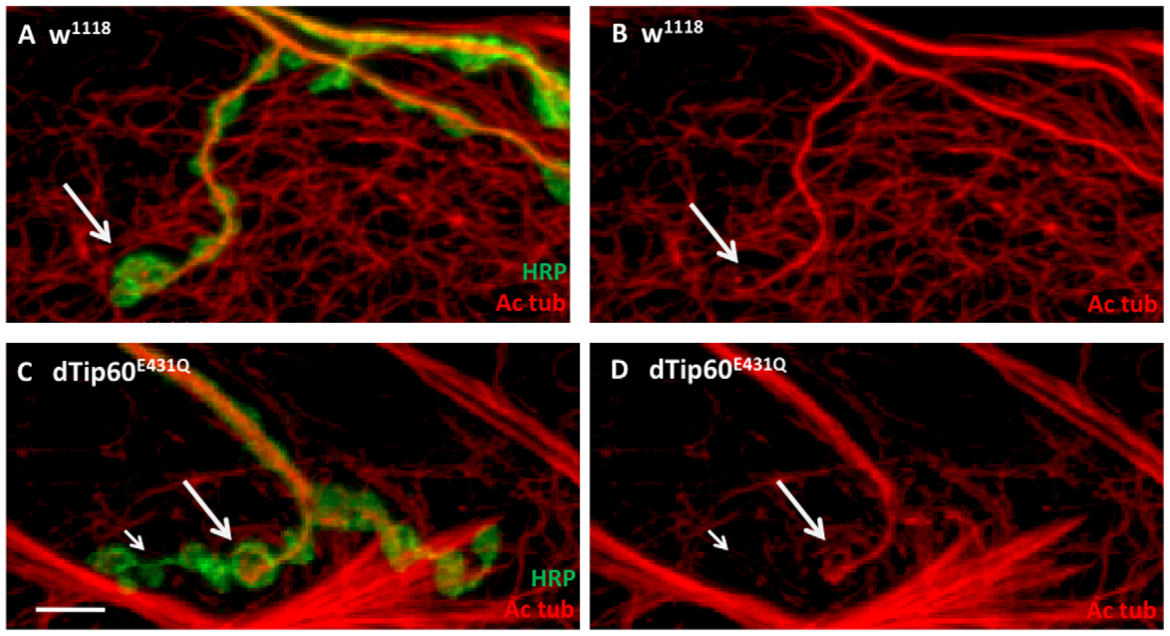
Loss of presynaptic dTip60 in the nervous system results in a reduction of acetylated microtubules in axons extending into terminal boutons. Confocal imaging analysis of boutons on muscles 6/7 at abdominal segment A4 immunohistochemically double labeled with HRP (green) and anti-acetylated α-tubulin antibody (red) that labels acetylated microtubules. (**A**) Merged image of HRP and acetylated α-tubulin antibody double labeled control w^1118^ larvae (**B**) acetylated α-tubulin antibody labeled control w^1118^ larvae (**C**) Merged image of HRP and acetylated α-tubulin antibody double labeled dTip60^E431Q^ larvae (D) acetylated α-tubulin antibody labeled dTip60^E431Q^ larvae. Of note, acetylated microtubule staining as a whole was decreased in dTip60^E431Q^ larvae (Figure 7 C and D). Large arrows represent acetylated microtubule staining in axons extending into boutons and small arrow represents their absence in more terminal boutons of dTip60^E431Q^ mutant larvae. In the analyses, w^1118^ genotype is represented by 31 larval preparations (n = 31) and dTip60^E431Q^ (n = 34). Scale bar is 5 um. Arrows show region of axon terminals where reduction of acetylated α-tubulin is consistently observed.

The increase in bouton number in dTip60^RNAi^ and dTip60^E431Q^ flies was accompanied by an excess of futsch-associated microtubule looping and splaying, suggesting that the boutons are undergoing rapid expansion as a result of being unstable. Microtubule dynamics play an important role in assisting bouton expansion and retraction, with acetylation of α-tubulin associated with microtubule stability and deacetylation associated with destabilization [45]. Though not important for cell survival, acetylation of microtubules has been shown to affect motor-dependant trafficking throughout neurons [46]. Recently, the histone acetyltransferase Elp3 was shown to directly acetylate microtubules in cortical neurons that appears to contribute to their migration and differentiation [47], and control bouton expansion at the *Drosophila* NMJ [18], however additional HATs that may also be involved in this process have not been identified. Thus, we asked whether acetylation of microtubules was reduced in response to dTip60 loss. To address this question, we performed immunohistochemical staining of NMJ boutons with anti-acetylated tubulin, which specifically recognizes α-tubulin in the acetylated state. In w^1118^ control NMJs, an acetylated microtubule network extended into the terminal boutons and was also present along the entire length of the axon (Figure 7 A and B). In contrast, examination of the NMJs in the dTip60^E431Q^ larvae revealed that acetylated microtubule staining as a whole was decreased (Figure 7 C and D). Closer observation revealed that acetylated microtubule staining was also consistently decreased in the axons extending into the terminal boutons, tapering off towards the end and particularly in those regions showing increased branching and bouton division, suggesting possible loss of stability due to reduced acetylation (Figure 7 C and D.) Of note, dTip60^RNAi^ expression did not show an observable effect on α-tubulin acetylation in the larvae, possibly due to the different mechanisms of dTip60 knockdown between the dTip60^RNAi^ versus dTip60^E431Q^ flies [19]. Additionally, overexpression of dTip60^WT^ also did not show an observable effect in tubulin acetylation level, bouton number or microtubule organization, possibly due to the fact that dTip60 has already reached its maximum function at the NMJ. These findings suggest that the dTip60^E431Q^ induced NMJ expansion phenotype may arise at least in part, from alteration in microtubule networks *via* loss of acetylation, either directly or indirectly by dTip60.

## Discussion

In this report, we investigate a role for dTip60 in synapse development and function using the highly characterized *Drosophila* neuromuscular junction as a model system. We show that dTip60 is highly concentrated at larval motor neuron synaptic boutons and is localized both pre and post synaptically, suggesting that dTip60 plays a role on both pre and postsynaptic sides of the NMJ in the differentiation of these neurons. In support of this possibility, we show that presynaptic depletion of dTip60 in the nervous system using GAL4 inducible fly lines dTip60^RNAi^ and HAT defective dTip60^E421Q^ results in a significant expansion of synaptic bouton number, indicating that this HAT negatively controls synaptic bouton formation and differentiation at the presynaptic side of the NMJ during the third instar larval stage. Interestingly, only type Is and not type Ib bouton numbers undergo significant expansion in response to dTip60 loss, supporting partial specificity in dTip60 function in certain bouton types. In addition to an increase in type Is boutons, we also observed an increase in the production of satellite boutons in response to dTip60 loss. Satellite bouton production has been observed for certain *Drosophila* proteins that affect synaptic plasticity, including overexpression of the *Drosophila* amyloid precursor protein APPL, a pan-neuronal protein implicated in Alzheimer's disease [48] and Shaggy, the *Drosophila* homolog of the glycogen synthase kinase 3 (GSK3βa kinase that negatively controls NMJ growth via microtubule cytoskeleton dynamics [29]. It is thought that satellite bouton formation is caused by an increased rate of sprouting and subsequent abnormal bouton differentiation [23], [48], thus implicating dTip60 in these processes. Remarkably, we find that there is an opposite effect on synaptic bouton number at the NMJ in response to postsynaptic Tip60 knockdown using both dTip60^RNAi^ and dTip60^E431Q^ HAT defective lines when compared to presynaptic knockdown, in that there is a significant reduction in bouton number, with virtually no formation of satellite boutons. Consistent with our finding, there are a number of NMJ proteins that contribute both pre and post-synaptically in the control of synaptic plasticity. For example, Discs Large (Dlg) is localized to type-1 glutamatergic synaptic terminals pre and post synaptically where it serves as a major scaffolding component at the larval NMJ [31], [49]. Spastin is another protein expressed at the NMJ that is concentrated both pre and post-synaptically with inappropriate localization having effects on microtubule stability that affect synaptic growth [50], [51]. The translational repressor Nanos also influences bouton number at the *Drosophila* NMJ from both the pre and postsynaptic sides [52]. Importantly, anterograde and retrograde modes of signaling are required at sites of synaptic contact and these signaling pathways play critical roles in the formation, differentiation and plasticity of synaptic connections [53]. For example, synaptic development at the *Drosophila* NMJ is dependent upon the bidirectional influence of wingless signaling on both pre and postsynaptic structures *via* distinct intracellular pathways [42]. Moreover, specific pre and postsynaptic levels of the cell adhesion molecule Fasciclin II are required to regulate appropriate synaptic growth *via* signaling through the fly homolog of amyloid precursor protein APPL [54]. Intriguingly, dTip60 has also been shown to be involved in APP signaling pathways *via* its complex formation with the C-terminal fragment of the amyloid precursor protein (APP) known as the APP intracellular domain (AICD) and linker protein Fe65 [22], [55], [56], [57]. Thus, based on the pre and postsynaptic staining pattern of dTip60, and the opposite phenotypic consequences on bouton number that results from its pre versus postsynaptic reduction, it is tempting to speculate that dTip60 may also be required for bi-directional signaling at the NMJ to regulate synapse formation and function.

Active zones are presynaptic specializations where synaptic vesicles accumulate and fuse to the plasma membrane in response to an action potential. Importantly, they are located in perfect opposition to the glutamate receptors on the postsynaptic side of the NMJ, and thus are a commonly used marker for both synapse number and functionality in synaptic transmission. Electron-dense cytoplasmic projections termed T-bars are often observed that extend from the active zone into the presynaptic cytoplasm and are believed to facilitate vesicle movement to this site to mediate neurotransmitter release [58], [59]. Bruchpilot (brp) is a scaffolding protein found in *Drosophila* at the active zones where it localizes to T-bars and is essential for synaptic transmission [38], [60]. Here, we show that although there was an increase in bouton number that resulted from presynaptic dTip60 loss, the boutons show a significant reduction in the abundance in the active zone synaptic function marker bruchpilot, suggesting a decrease in their neurotransmitter function and linking dTip60 HAT activity to this process. As postsynaptic reduction of dTip60 results in a significant reduction of 1 s boutons at the NMJ, it will be important for future studies to investigate whether these remaining boutons are also negatively impacted, in order to determine whether there is postsynaptic contribution of dTip60 on Bruchpilot expression and bouton function. Intriguingly, the precise regulation of BRP mediated neurotransmission process has been shown to be critical for higher order nervous system function that includes learning, memory and cognition [61]. Thus, it is tempting to speculate that dTip60 HAT activity plays a critical role in the control of neurotransmission important for these processes.

Tip60 HAT activity has been implicated in the age-related neurodegenerative disorder Alzheimer's disease (AD) *via* its HAT dependent complex formation with the C-terminal fragment of the amyloid precursor protein (APP) known as the APP intracellular domain (AICD) and linker protein Fe65 [22], [55], [56], [57]. The association of these proteins, termed the AFT complex, epigenetically control the transcriptional regulation of target genes involved in neuronal function [22], [57]. Interestingly, the *Drosophila* NMJ phenotype that results from presynaptic inhibition of Shaggy kinase activity [29] is very similar to our dTip60 HAT presynaptic depletion mutant NMJ phenotype, which includes expansion of synaptic bouton number, and an increase in satellite bouton formation that is accompanied by an excess of Futsch stained microtubule loops. Shaggy is thought to function in the negative regulation of bouton expansion *via* phosphorylation of the MAP1B microtubule binding protein Futsch, thus inhibiting its action in promoting formation of the microtubule loops that are associated with stable bouton formation [41], [42]. Intriguingly, Gsk3β (the mammalian shaggy homolog) has been shown to be a direct transcriptional target of the AFT complex, where Tip60 HAT activity is required for the epigenetic control of Gsk3β transcriptional activation [56]. Based on these findings, the NMJ overgrowth phenotype shared by Tip60, Shaggy and APPL (*Drosophila* APP homolog) mutants may suggest that these proteins are involved in overlapping transcriptional regulatory pathways that could potentially be involved in the synaptic defects observed in early AD progression. Additionally, as we observed a decrease in bruchopilot immunostaining and an increase of Futsch stained microtubule loops in our dTip60 mutant larvae, it will be important in future studies to determine whether these neuronal marker genes, as well as *shaggy* are direct transcriptional targets of dTip60.

Alternatively, dTip60 may also directly regulate synaptic microtubule architecture independent of epigenetic based transcriptional mechanisms. For example, we found that α-tubulin acetylation levels of microtubules specifically extending into the terminal synaptic boutons are reduced in response to dominant negative HAT defective dTip60^E431Q^ overexpression, but not dTip60^RNAi^ induced knockdown. One possible explanation for this observation is that in the dTip60^E431Q^ mutant, there is competition for acetylation of tubulin between the wild-type dTip60 and dTip60^E431Q^ protein, whereas in the RNAi induced knockdown, there is still enough residual endogenous dTip60 protein available for acetylation function. Consistent with this interpretation, the decrease in tubulin acetylation levels we observe in the dTip60^E431Q^ larvae is subtle, possibly indicative of the presence of endogenous dTip60 protein and/or other endogenous factors that can still acetylate tubulin. One such endogenous factor may be the HAT Elp3, an acetylase shown to directly acetylate microtubules in cortical neurons that contributes to their migration and differentiation [47]. These studies demonstrate that loss of Elp3 in cultured projection neuronal cells leads to severe defects in axonal branching [47], [62], [63]. Intriguingly, our previous studies investigating a role for Elp3 in synaptic plasticity demonstrate a very similar phenotype to our dTip60 mutants, in that bouton expansion is significantly increased in response to presynaptic RNAi induced Elp3 loss [47]. Thus, it will be important to determine whether expression, localization and activity of Elp3 is affected at the NMJ in our dTip60 mutant lines. We do note that the link we observe between dTip60 loss and reduction of α-tubulin acetylation levels of microtubules at the NMJ is correlative in nature. Therefore, it will also be important to decipher whether dTip60 acts indirectly or directly to acetylate tubulin on specific residues and whether this acetylation directly impacts specific biological processes such as microtubule architecture and/or influences interaction with microtubule binding proteins such as Futsch. Interestingly, here we observe that although acetylation of tubulin is not significantly decreased in response to dTip60^RNAi^ induced knockdown, pupal lethality still occurs, suggesting that the acetylation of tubulin cannot be specifically linked to nervous specific dTip60 induced lethality. These results are consistent with studies on HDAC6 knockout mice, a class II HDAC known to target K40 acetylation of α-tubulin, demonstrating that although these mice display a significant increase in tubulin acetylation in the brain, this phenomenon does not rescue disease progression in a mouse model of Huntington's disease [64], [65], [66].

The emerging hypothesis that age-related aberrant changes of specific acetylation marks in chromatin in the adult brain lead to gene misregulation that drives cognitive decline and specifically, memory impairment in the elderly, underscores the crucial role HATs play in cognitive ability [11], [12]. A role for HAT activity in learning and memory is not unprecedented, with the HAT CBP implicated as a critical component of memory consolidation via the site specific histone acetylation of chromatin in the brain that in turn, regulates long-term transcriptional changes associated with long-lasting forms of neuronal plasticity [67]. Importantly, recent studies show that synaptic activity can influence such CBP associated histone acetylation marks, thus providing a mechanism for how external environmental stimuli can influence behavioral dependant synaptic plasticity that is linked to memory. In this model of synaptic activity-dependent epigenetic regulation, synaptic input and depolarizing stimuli cause an increase in Ca2+ intracellular levels via specific Ca2+ channels, thus activating certain kinases to phosphorylate CBP which is thought to promote its recruitment to chromatin [5], [68]. CBP mediated specific histone acetylation marks cooperate with additional epigenetic modifications to induce chromatin structural changes that regulate gene expression of synaptic activity-dependant genes. In support of this model, genome-wide screens of mouse cortical neurons using ChIP-seq have shown that membrane depolarization markedly enhances CBP recruitment to such enhancers [69]. Intriguingly, neural activity can also modulate chromatin acetylation by regulating the shuttle of class II HDACs in and out of the nucleus in hippocampal neurons [70]. As dTip60 has also been shown to shuttle between nuclear and cytoplasmic cellular compartments, with misregulation of this process associated with prostate cancer [71], [72], it is tempting to speculate that cellular localization of Tip60 influences synaptic plasticity in a manner similar to certain HDACs. Our findings causatively linking Tip60 HAT activity to the control of synaptic bouton formation and function, in conjunction with future studies deciphering the mechanisms of how Tip60 controls such processes, should further our understanding of epigenetic mechanisms underlying synaptic plasticity and memory formation in neurodevelopment, age-related cognitive decline and neurodegenerative disorders.

## Materials and Methods

### Drosophila Stocks

All fly lines used were raised under standard conditions at 25°C on standard yeasted drosophila media (Applied Scientific Jazz Mix Drosophila Food, Thermo Fisher Scientific, Waltham, MA, USA). The pan-neuronal driver elav^c155^ was obtained from Bloomington Stock Center (Bloomington, IN). *P{pUAST}/P{pUAST}* fly line containing dTip60^WT^ (independent lines A and B), dTip60^E431Q^ (independent lines A and B) and dTip60^RNAi^ (line A) were created as described [32]. Muscle specific GAL4 drivers MEF-2 and TWI;MEF2 was a generous gift from Xianmin Zhu. As the elav^c155^ transgene is located on the X chromosome, homozygous female elav^c155^ flies were crossed to homozygous male dTip60^WT^, dTip60^E431Q^ and dTip60^RNAi^ fly lines for all studies. All experimental crosses were carried out at 25°C.

#### Immunohistochemistry

3^rd^ instar larvae were filleted in HL-3 saline, pH 7.2 and pinned out on Sylgard dishes with guts removed. Larvae progeny of either sex were used from elav^c155^ lines crossed to dTip60^WT^ and dTip60^E431Q^ lines. For the dTip60^RNAi^ line, as this transgene is located on the X chromosome, only female larvae progeny were selected for analysis. Fillet preparations were fixed in 3.5% paraformaldehyde for 30 minutes, followed by 3 washes in PBS and one with 1× PBS with 0.1% Triton. The larvae were then incubated overnight in primary antibody overnight at 4°C. After 6 washes in PBS-T (1× phosphate buffered saline +0.1% Triton), fillets were incubated in 2° antibody for 1 hour, washed twice in 1× PBS-T, once in 1× PBS, then mounted onto slides in Vectashield antifade mounting media (Vector laboratories, Burlingame CA). Confocal microscopy was performed using Olympus Microscope with fluoview software.

#### Antibodies

The primary antibodies, anti-DCSP-3 (1G12), anti-DLG (4F3), anti-Futsch (22C10) and anti-nc82 antibodies were all obtained from Developmental Studies Hybridoma Bank, DSHB (University of Iowa, Iowa City, IA). Each of these antibodies was diluted in block (2%BSA, 5%NGS in 1XPBS-T) and used at a dilution of 1∶10. Anti-Syt (*Littleton et al.*, 1993) was a generous gift from Hugo Bellen and was used at 1∶5000 dilution. The primary mouse monoclonal anti-acetylated tubulin antibody (clone 6-11B-1) was obtained from Sigma-Aldrich (St. Louis, MO) and used at a dilution of 1∶1000 in block. Polyclonal rabbit anti-dTip60 antibody (Lorbeck et al., 2011) was used at a dilution of 1∶100. The HRP Antibody was obtained from Jackson Immunoresearch Laboratories Inc. and used at a dilution of 1∶25.

#### Imaging and Analysis

Larval NMJ's were imaged on a Olympus (Germany) FV1000 laser scanning confocal microscope. All images were captured using same constant confocal gain settings. Images were acquired as a Z-stack and then rendered as a maximum projection. Individual entire muscle 6/7 NMJ synapses were optically sectioned (0.2 um; series of 15 to 20 sections per synapse). A 2D projection was generated that projected the pixel of maximal intensity found within the series of sections. Quantitative analysis of fluorescence intensities was done using FV1000 software. For comparison between genotypes, all samples were processed simultaneously and imaged using identical microscopic acquisitions parameters. All images were also corrected for any background before any intensity measurements. Student's T-test was used for all statistical analysis.

## Supporting Information

Figure S1 **Expression levels of dTip60 at the larval NMJ.** Figure (**A**) represents confocal imaging analysis of control w^1118^ larval boutons on muscles 6/7 at abdominal segment A4 immunohistochemically stained with dTip60 antibody (blue). Figure (**B**) dTip60 (blue) showing the presence of presynaptic dTip60 and absence of postsynaptic dTip60 localization due to RNAi postynaptic knockdown using the muscle specific MEF-2 GAL4 driver, demonstrating efficacy of dTip60 knockdown. Figure (**C and D**) showing dTip60 (blue) postsynaptic overexpression of the dTip60^E431Q^ and dTip60^WT^ transgenes using muscle specific driver MEF-2. In the analyses, w^1118^ genotype is represented by 18 larval preparations (n = 18), dTip60^WT^ (n = 18), dTip60^RNAi^ (n = 17) and dTip60^E431Q^ (n = 20). Scale bar 10 um.
(TIF)

Figure S2 **Total number of boutons in control fly lines.** (A) Flies homozygous for each of the genotypes indicated were crossed to flies homozygous for the nervous system elav^C155^ pan-neuronal GAL4 driver, and staged third instar progeny larvae were collected. Confocal imaging analysis of larval boutons on muscles 6/7 at abdominal segment A4 immunohistochemically stained with anti-HRP (green) that labels the entire presynaptic membrane. Histogram represents the total bouton number for each genotype indicated. Histogram (**B**) represents the total bouton number for each of the UAS-transgenic larvae indicated in the absence of a GAL4 driver. Genotypes are each represented by 15 larval preparations (n = 15).
(TIF)

Figure S3 **Relative number of boutons to futsch stained loops.** Histogram represents the relative number of boutons to futsch stained loops for each of the genotypes indicated calculated from data presented in Figure 3.
(TIF)
