# Supplementary figures and images for: dTip60 HAT Activity Controls Synaptic Bouton Expansion at the Drosophila Neuromuscular Junction
Source: PLoS One. 2011 Oct 27;6(10):e26202. doi: 10.1371/journal.pone.0026202 (PMC3203119; doi:10.1371/journal.pone.0026202)

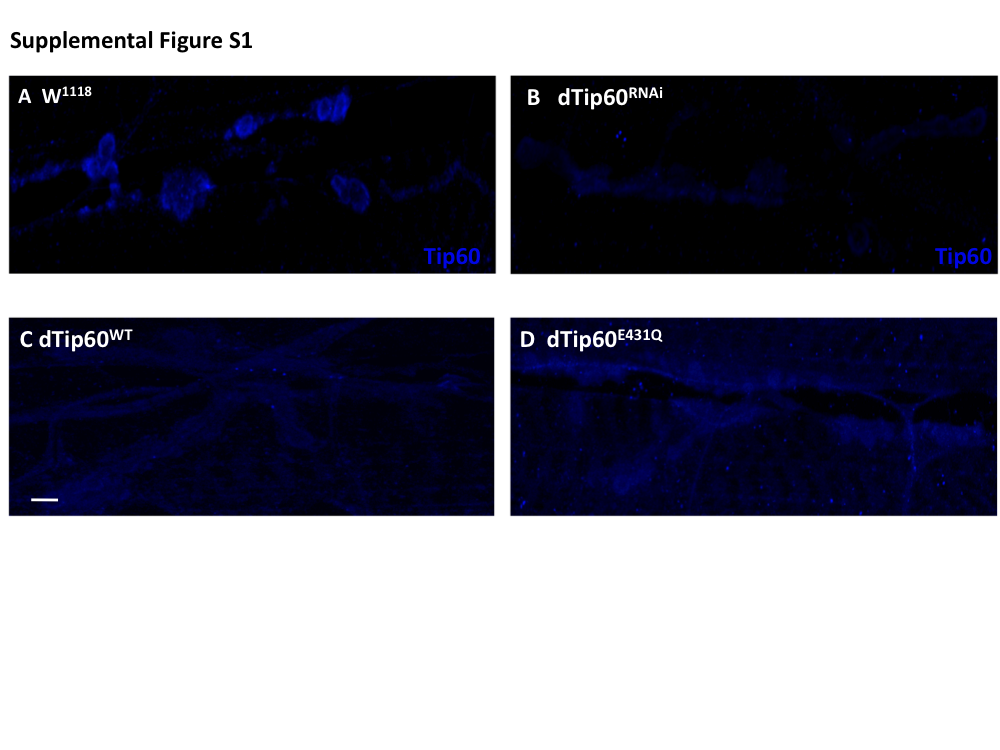

Supplement: Figure S1 — Expression levels of dTip60 at the larval NMJ. Figure (A) represents confocal imaging analysis of control w1118 larval boutons on muscles 6/7 at abdominal segment A4 immunohistochemically stained with dTip60 antibody (blue). Figure (B) dTip60 (blue) showing the presence of presynaptic dTip60 and absence of postsynaptic dTip60 localization due to RNAi postynaptic knockdown using the muscle specific MEF-2 GAL4 driver, demonstrating efficacy of dTip60 knockdown. Figure (C and D) showing dTip60 (blue) postsynaptic overexpression of the dTip60E431Q and dTip60WT transgenes using muscle specific driver MEF-2. In the analyses, w1118 genotype is represented by 18 larval preparations (n = 18), dTip60WT (n = 18), dTip60RNAi (n = 17) and dTip60E431Q (n = 20). Scale bar 10 um. (TIF) [file pone.0026202.s001.tif]

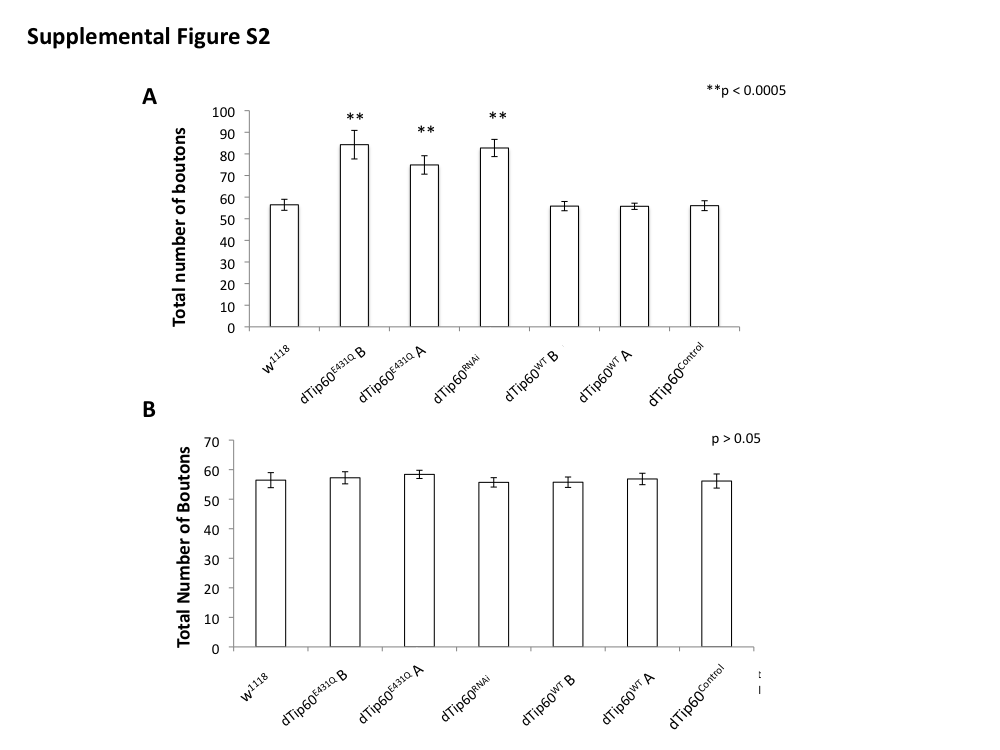

Supplement: Figure S2 — Total number of boutons in control fly lines. (A) Flies homozygous for each of the genotypes indicated were crossed to flies homozygous for the nervous system elavC155 pan-neuronal GAL4 driver, and staged third instar progeny larvae were collected. Confocal imaging analysis of larval boutons on muscles 6/7 at abdominal segment A4 immunohistochemically stained with anti-HRP (green) that labels the entire presynaptic membrane. Histogram represents the total bouton number for each genotype indicated. Histogram (B) represents the total bouton number for each of the UAS-transgenic larvae indicated in the absence of a GAL4 driver. Genotypes are each represented by 15 larval preparations (n = 15). (TIF) [file pone.0026202.s002.tif]

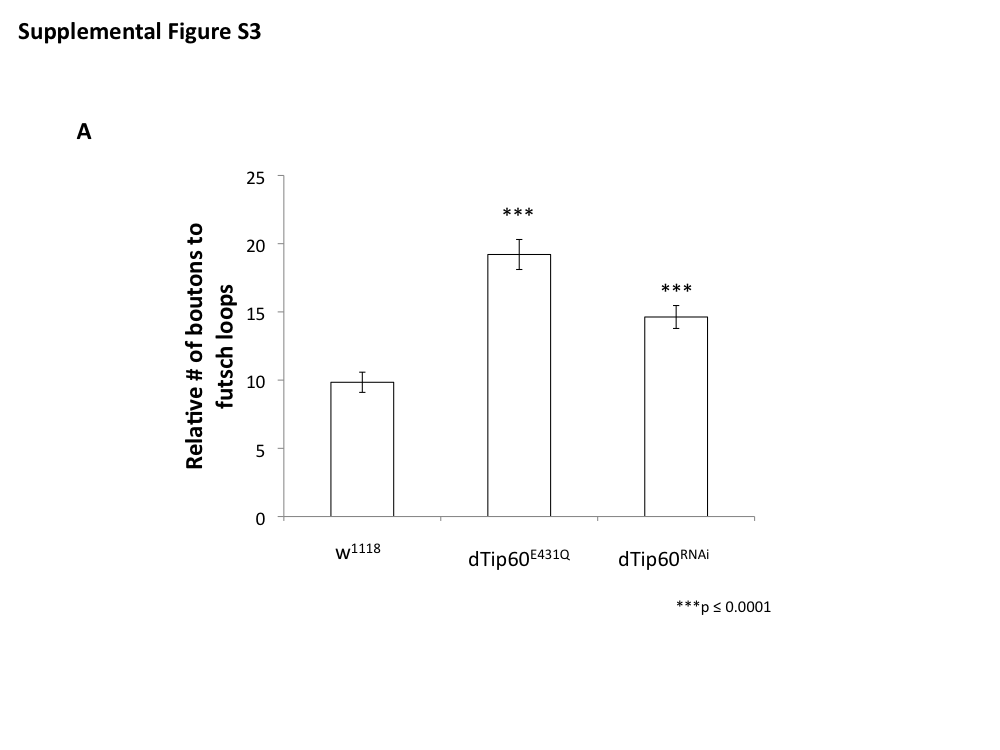

Supplement: Figure S3 — Relative number of boutons to futsch stained loops. Histogram represents the relative number of boutons to futsch stained loops for each of the genotypes indicated calculated from data presented in Figure 3. (TIF) [file pone.0026202.s003.tif]
